# Supplementary material for: Antibiotic prophylaxis for open lower extremity fractures: a comparative propensity-scored matched analysis of cefazolin vs. piperacillin-tazobactam
Source: Arch Orthop Trauma Surg. 2026 Apr 29;146(1):166. doi: 10.1007/s00402-026-06333-0 (PMC13128684; doi:10.1007/s00402-026-06333-0)
Supplement: Supplementary file 1 — Supplementary Material 1 [file 402_2026_6333_MOESM1_ESM.docx]

**Antibiotic Prophylaxis for Open Lower Extremity Fractures: A Comparative Propensity-Scored Matched Analysis of Cefazolin vs Piperacillin-Tazobactam**

**ABSTRACT** *(299 words)*

**Introduction**

Optimal antibiotic prophylaxis for open fractures remains controversial, particularly regarding whether broader-spectrum regimens offer clinical advantages over cefazolin monotherapy. Despite guideline recommendations supporting first-generation cephalosporins for most open fractures, many centers routinely administer piperacillin-tazobactam. This study aims to evaluate differences in postoperative outcomes between cefazolin and piperacillin-tazobactam in lower extremity open fractures across all Gustilo-Anderson types.

**Materials and Methods**

This retrospective cohort study was performed using the TriNetX database to identify adult patients with Gustilo-Anderson type I-III lower extremity open fractures who received either cefazolin or piperacillin-tazobactam. 1:1 propensity score matching controlled for age, sex, demographics, and relevant comorbidities. Outcomes were assessed at 90 days and 1 year using risk ratios (RR) with corresponding 95% confidence intervals.

**Results**

A total of 47,692 patients met inclusion criteria prior to matching. After matching, 1,527 patients remained in each treatment group for the combined type I/II/III cohort and similar matched pairs were obtained for type I/II and type III subgroups. At 90 days, cefazolin was associated with significantly lower rates of surgical site infection (RR 0.569), osteomyelitis (RR 0.292), sepsis (RR 0.244), reoperation (RR 0.474), readmission (RR 0.518), thromboembolic events (RR 0.480), AKI (0.448), and mortality (RR 0.208) across most analyses. Nonunion/malunion rates were similar between groups for type I/II and the combined cohort (p > 0.05), but higher among type III fractures treated with cefazolin (RR1.933). At 1 year, cefazolin was associated with significantly lower reoperation (RR 0.564), implant removal (RR 0.585), and mortality (RR 0.298) across all analyses, with persistently higher nonunion/malunion risk in type III fractures (RR 1.929).

**Conclusions**

Cefazolin was associated with comparable or superior outcomes to piperacillin-tazobactam for most postoperative complications following lower extremity open fractures. These findings support current guideline-aligned stewardship practices and question the routine use of broader-spectrum prophylaxis, particularly outside specific contamination scenarios.

**Key Words**

- **Open fractures**
- **Antibiotic prophylaxis**
- **Cefazolin**
- **Piperacillin-tazobactam**

**Level of Evidence:** III – Retrospective Comparative Study

**INTRODUCTION**

Open fractures remain a significant clinical challenge, with high risks of infection, delayed union, and long-term morbidity if not managed appropriately. Standard treatment includes timely surgical debridement, stabilization, and early administration of prophylactic antibiotics [1]. Among these therapeutic measures, numerous studies have underscored the importance of prompt antibiotic delivery as a key driver of outcomes [1–3].

For decades, the Gustilo-Anderson classification system has served as the foundation for both surgical and medical decision-making in open fractures [4]. This system stratifies injuries based on wound size, contamination, and soft tissue damage: Type I injuries involve wounds <1 cm with minimal contamination, Type II includes wounds 1–10 cm without extensive soft tissue injury, and Type III encompasses wounds >10 cm with significant soft tissue damage, contamination, or vascular compromise. Traditional antibiotic guidelines, including those by the American Academy of Orthopaedic Surgeons, have recommended first-generation cephalosporins like cefazolin for Type I and II injuries, with the addition of aminoglycosides (e.g., gentamicin) for Type III fractures, and further agents like penicillin or fluoroquinolones for specific exposures (e.g., farm or water-related injuries) [4–6].

Despite these longstanding recommendations, real-world practice often deviates. In some trauma centers, broad-spectrum agents such as piperacillin-tazobactam are routinely used for all open fractures, regardless of severity or contamination [7]. The rationale behind these expanded regimens typically centers on concern for resistant or polymicrobial organisms in high-energy injuries, as well as a perceived need for “maximum coverage” in severely contaminated wounds [8]. Surgeons in these settings may view broader-spectrum antibiotics as a proactive way to reduce infection risk in complex trauma, particularly where the wound environment is uncontrolled or difficult to characterize [8]. Additionally, some trauma centers opt for standardized antibiotic protocols regardless of Gustilo-Anderson type for logistical reasons, choosing to maximize adherence and minimize time to antibiotic administration [3].

At the same time, emerging data has called into question whether such broad-spectrum approaches meaningfully improve outcomes. Several recent studies have found no added benefit in infection prevention or long-term complications when extended-spectrum agents are used over cefazolin alone, even in high-grade injuries [7, 9–11]. Lin et al., for example, found no significant difference in infection rates between cefazolin monotherapy and combination regimens in open fractures of all Gustilo-Anderson grades [11]. These findings raise important questions about the necessity, safety, and cost-effectiveness of routine polypharmacy in this setting.

This ongoing divergence between guideline-based recommendations and current clinical practice highlights a critical gap in trauma care. While timing of antibiotic administration remains universally emphasized, there is still no consensus on the optimal prophylactic regimen for different open fracture scenarios [2, 3]. The purpose of this study is to compare postoperative outcomes associated with cefazolin versus piperacillin-tazobactam in adults with Gustilo-Anderson type I-III open lower extremity fractures. The study hypothesis is that cefazolin is associated with lower rates of postoperative infectious complications compared with piperacillin-tazobactam. In an era of increasing antibiotic resistance, cost containment, and antimicrobial stewardship, this study seeks to clarify which antibiotics impart the greatest therapeutic advantage for open fracture prophylaxis.

**METHODS**

TriNetX is a global health research database that aggregates electronic medical record data from over 120 healthcare organizations (HCOs), encompassing more than 250 million patients globally. The platform provides access to de-identified, retrospective clinical data, including diagnoses, procedures, medications, laboratory values, vital signs, genomics, and patient demographics. In compliance with HIPAA §164.514(b)(1), all data are de-identified and presented in aggregate form, exempting associated studies from Institutional Review Board (IRB) review. Participating institutions contribute data in accordance with their own institutional privacy standards and are granted access to the platform’s suite of analytics, visualization tools, and research infrastructure in return.

This study was run on the TriNetX Research Network, which includes 102 HCOs. The database was retrospectively queried in July 2025, using standardized coding systems including Current Procedural Terminology (CPT), International Classification of Diseases, 10^th^ Revision (ICD-10), and RxNorm. Patients aged ≥18 years were included if they presented with an initial encounter for an open fracture of the lower extremity and received either cefazolin or piperacillin-tazobactam during their initial treatment. This approach was used to preferentially identify patients undergoing initial evaluation and treatment for their open fracture rather than follow-up encounters related to prior injuries. To isolate the effect of each antibiotic regimen, patients who received concomitant administration of other broad-spectrum or gram-positive agents on the same day as the index fracture and antibiotic were excluded. Specifically, patients receiving cefazolin were excluded if they also received piperacillin-tazobactam, ceftriaxone, vancomycin, or gentamycin on the same day. Similarly, patients in the piperacillin-tazobactam cohort were excluded if they received cefazolin, vancomycin, ceftriaxone, or gentamycin on the same day. This approach was used ot ensure that each cohort reflected monotherapy exposure at the time of initial prophylaxis.

To control for baseline differences between treatment groups, propensity score matching (PSM) was performed using 1:1 greedy nearest neighbor matching with a caliper of 0.1. Matching variables included patient age, sex, race, ethnicity, smoking status, and relevant comorbidities (diabetes, peripheral vascular disease, chronic kidney disease, and obesity). Adequate covariate balance was assessed using standardized mean differences (SMD), with values of < 0.100 considered acceptable [12]. Three separate analyses were conducted based on the Gustilo-Anderson classification of open lower extremity (LE) fractures: (1) Type I/II, (2) Type III, and (3) Combined Type I/II/III fractures using respective ICD-10 codes. A flow diagram illustrating cohort selection is shown in **[Fig. 1]**.

Postoperative outcomes were assessed at 90-day and 1-year intervals. The primary outcome was rates of surgical site infections (SSI) at 90 days. Secondary 90-day outcomes included osteomyelitis, sepsis, reoperation, hospital readmission, emergency department (ED) visits, thromboembolic events (including deep vein thrombosis or pulmonary embolism), acute kidney injury, nonunion/malunion, and mortality. At one-year, outcomes observed included rates of nonunion/malunion, reoperation, implant removal, and mortality. Descriptive statistics were reported as means with standard deviations (SD) for continuous variables and proportions for categorical variables. Risk ratios (RR) with corresponding 95% confidence intervals (CIs) were calculated for all outcomes. A two-tailed p-value < 0.05 was used to define statistical significance. All statistical analysis was conducted within the TriNetX platform.


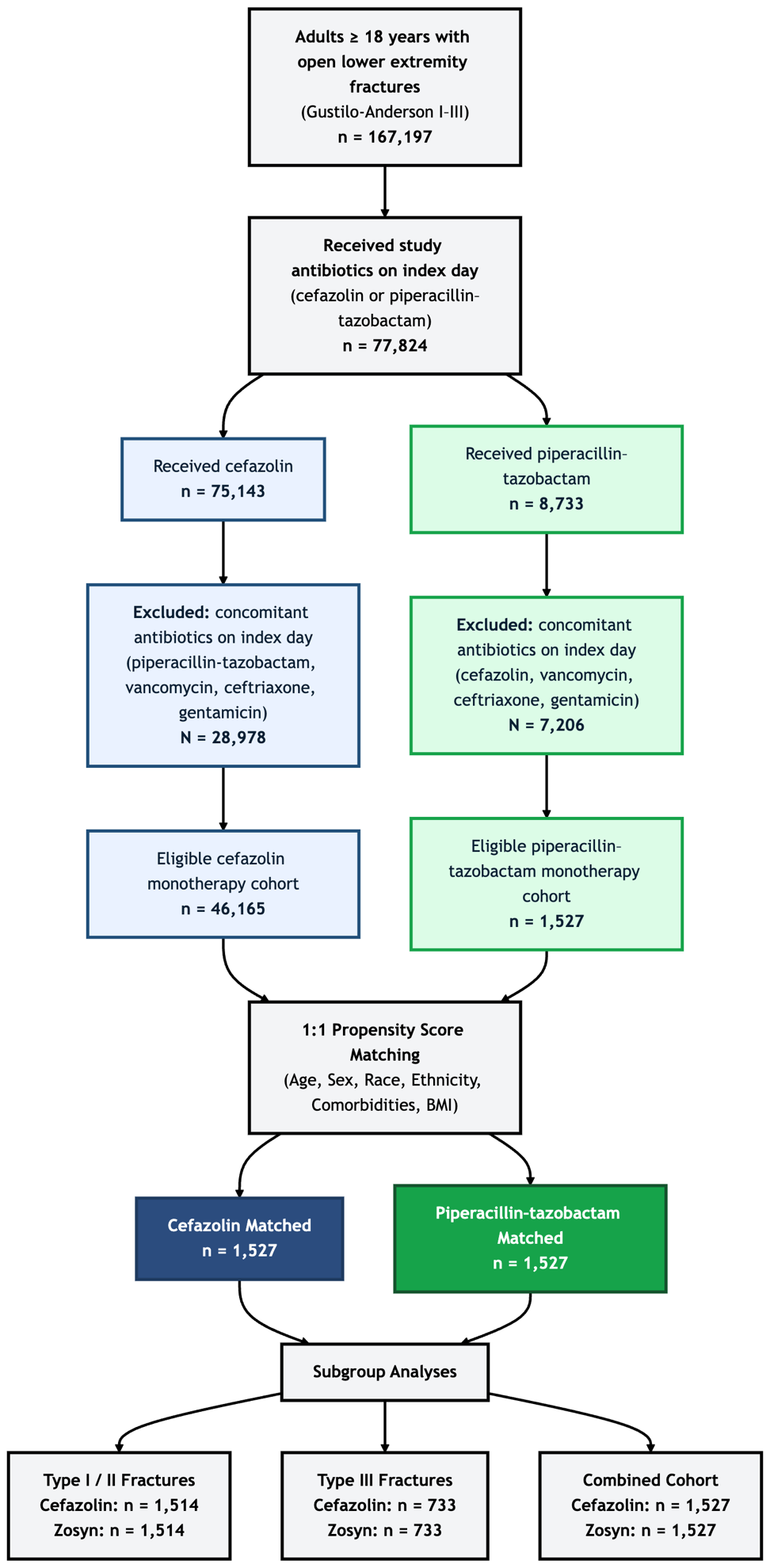


**Fig. 1** Cohort selection flow diagram for adult patients with open lower extremity fractures identified in TriNetX

**RESULTS**

**Patient Demographics and Baseline Characteristics**

A total of 47,692 adult patients with Gustilo-Anderson type I, II, or III open lower extremity fractures met inclusion criteria across all analyses. For the combined cohort (types I/II/III), 46,165 patients received cefazolin and 1,527 received piperacillin-tazobactam prior to matching. After 1:1 propensity score matching, 1,527 patients remained in each treatment group with no statistically significant differences in age, race, or comorbidities (SMD < 0.1) **[Table 1] [Fig. 1]**.

In subgroup analyses, 45,122 patients with type I/II fractures were treated with cefazolin and 1,514 with piperacillin-tazobactam. This yielded 1,514 matched pairs **[Table 2] [Fig. 1]**. For type III fractures, 11,939 received cefazolin and 735 received piperacillin-tazobactam, with 733 matched pairs retained for analysis **[Table 3] [Fig. 1]**. Across all three analyses, baseline characteristics including age, sex, race, ethnicity, and major comorbidities were well-balanced after matching for all covariates (SMD < 0.10). Baseline demographics pre- and post-propensity score matching are reported in full in **[Tables 1-3]**. Due to the dynamic nature of the TriNetX platform, which reflects real-time updates from participating HCOs, total patient counts may vary slightly between separate analyses, even when similar query parameters are used at the same time. This is a known limitation of real-time federated datasets and does not affect the integrity of the matching or subsequent outcome comparisons presented [13].

**Primary Outcome**

In the combined type I/II/III cohort, 90-day surgical site infection occurred less frequently in patients treated with cefazolin when compared to those receiving piperacillin-tazobactam (RR 0.569, 95% CI: 0.428 – 0.757). SSI rates were similarly lower in type I/II fractures (RR 0.475, 95% CI: 0.351 – 0.645). Although the type III subgroup trended toward fewer SSIs with cefazolin (RR 0.697, 95% CI: 0.485 – 1.001), the result did not meet statistical significance (p = 0.051) **[Table 4]**.

**Short Term Clinical Outcomes**

Rates of osteomyelitis were also significantly reduced in all fracture types, including type I/II (RR 0.352, 95% CI: 0.251 – 0.495), type III (RR 0.407, 95% CI: 0.251 – 0.662), and combined type I/II/III (RR 0.292, 95% CI: 0.202 – 0.422). Cefazolin was also associated with significantly lower sepsis rates across all fracture subgroups (RR 0.244, 95% CI: 0.148 – 0.400). Reoperation risk within 90 days was significantly reduced with cefazolin among type I/II (RR 0.381, 95% CI: 0.239 – 0.606) and in the combined cohort (RR 0.474, 95% CI: 0.301 – 0.745). The type III cohort demonstrated a similar trend but did not reach statistical significance (p > 0.05). Furthermore, readmission was significantly less frequent with cefazolin across all fracture subgroups (RR 0.518, 95% CI: 0.479 – 0.560). Thromboembolic events (DVT or PE) were significantly lower among type I/II fractures (RR 0.442, 95% CI: 0.310 – 0.631) and in the combined type I/II/III cohort (RR 0.480, 95% CI: 0.341 – 0.674), with a non-significant reduction in type III (RR 0.714, 95% CI 0.469 – 1.089). Acute kidney injury (AKI) risk was similarly significantly lower with cefazolin for type I/II fractures (RR 0.596, 95% CI: 0.431 – 0.823) and the combined cohort (RR 0.448, 95% CI: 0.315 – 0.637), but not significant in type III fractures. There were no significant differences observed between groups in emergency department visits. Nonunion/malunion rates did not differ significantly between groups for patients type I/II fractures (RR 0.710, 95% CI: 0.413 – 1.220, p > 0.05) or the overall type I/II/III cohort (RR 0.680, 95% CI: 0.369 – 1.254, p > 0.05). In contrast, among patients with type III fractures, Cefazolin was associated with significantly higher nonunion/malunion rates compared with piperacillin-tazobactam (RR 1.933, 95% CI: 1.045 – 3.577). Finally, cefazolin was associated with reduced 90-day mortality in all 3 subgroups (RR 0.208, 95% CI: 0.120 – 0.362) **[Table 4]**.

**Long-Term Clinical Outcomes**

At one year, reoperation remained significantly less frequent among type I/II fractures (RR 0.450, 95% CI: 0.342 – 0.594), type III fractures (RR 0.667, 95% CI: 0.486 – 0.914), and the combined cohort (RR 0.564, 95% CI: 0.439 – 0.725). There was no significant difference in nonunion/malunion rates between cefazolin and piperacillin-tazobactam for patients with type I/II fractures (RR 0.846, 95% CI: 0.570 – 1.257, p > 0.05) or for the combined type I/II/III cohort (RR 0.681, 95% CI: 0.437 – 1.061, p > 0.05). In contrast, among patients with type III fractures, nonunion/malunion occurred significantly more frequently in the cefazolin group compared with piperacillin-tazobactam (RR 1.929, 95% CI: 1.235 – 3.011). Implant removal occurred significantly less often with cefazolin across all analyses: type I/II (RR 0.652, 95% CI: 0.451 – 0.942), type III (RR 0.458, 95% CI: 0.280 – 0.751), and combined type I/II/III (RR 0.585, 95% CI: 0.413 – 0.830). The survival advantage persisted at 1 year. Mortality was lower with cefazolin among type I/II fractures (RR 0.370, 95% CI: 0.239 – 0.572), type III fractures (RR 0.558, 95% CI: 0.342 – 0.910), the combined cohort (RR 0.298, 95% CI: 0.192 – 0.463) **[Table 5]**.

**DISCUSSION**

Open lower extremity fractures carry a substantial risk of infection and other postoperative complications, prompting debate regarding optimal choice of prophylactic antibiotics. This large, propensity score-matched analysis of over 47,000 patients with lower extremity open fractures showed that cefazolin monotherapy was associated with lower rates of several postoperative complications when compared to piperacillin-tazobactam. Across most outcomes at both 90 days and one year, narrow-spectrum prophylaxis with cefazolin was associated with similar or lower complication rates compared with broader-spectrum therapy. Specifically, the cefazolin group experienced lower rates of surgical site infections, osteomyelitis, sepsis, reoperation, readmission, thromboembolic events, AKI, and mortality within the 90-day postoperative period. With regards to long-term outcomes, cefazolin patients experienced lower rates of reoperation, implant removal, and mortality at one year post-operatively. Although nonunion/malunion rates were similar for type I/II fractures and the combined cohort, rates were higher among type III fractures receiving cefazolin monotherapy.

While cefazolin was associated with lower rates of mortality and systemic complications, these outcomes are also strongly influenced by overall injury severity, institutional care pathways, and physiologic insult in addition to antibiotic selection. Therefore, the findings of this study should be interpreted cautiously, as unmeasured differences in trauma severity may partially account for the magnitude of observed associations. As such, these findings should be interpreted as associations rather than evidence that antibiotic spectrum alone directly reduces systemic complications. Overall, these findings challenge the longstanding assumption that expanded gram-negative and anaerobic coverage improves outcomes following high-energy traumatic injuries, while also demonstrating fracture-severity-specific nuances that merit further study.

These results have important implications for clinical practice and antibiotic stewardship. The routine use of broad-spectrum agents in open fractures has traditionally been justified by concerns about polymicrobial contamination, particularly in high-energy type III fractures [6]. However, the present findings suggest that broader coverage does not necessarily translate to improved clinical outcomes and may expose patients to avoidable risks, including renal injury and increased healthcare utilization. The findings of this study showing that narrow-spectrum, guideline-aligned prophylaxis may be sufficient for the majority of open fractures by demonstrating comparable or superior outcomes.

The results of this study align with several prior studies that have investigated the proposed benefits of broad-spectrum regimens in open fracture prophylaxis. In a multicenter, retrospective cohort study, Pantanwala et al. reported no reduction in infectious complications with expanded gram-negative coverage in type III open fractures when compared to cefazolin monotherapy [10]. Additionally, O’Connell et al. found in a single-center retrospective cohort study that piperacillin-tazobactam prophylaxis did not improve infection rates compared to first-generation cephalosporin with or without an aminoglycoside in type III open fractures [7]. More recent data by Lin et al. demonstrated comparable postoperative infectious risk in patients receiving cefazolin alone and those with extended polymicrobial coverage in patients with type I, II, or III open fractures [11]. The current study aligns with these findings and extends them by using a vastly larger, multicenter cohort with rigorous matching to reduce confounding. The magnitude and consistency of the infection- and complication-related risk reductions observed with cefazolin reinforce the accumulating evidence that broader antimicrobial coverage offers limited benefit in clinical practice.

Several biologic mechanisms may plausibly explain the observed differences between cefazolin and piperacillin-tazobactam in this study. Cefazolin is a first-generation cephalosporin with potent activity against the gram-positive organisms that are most often implicated in post-traumatic wound complications, including *Staphylococcus aureus* and *Streptococci* [14]. Cefazolin achieves a high and sustained concentration in serum, skeletal muscle, and bone, which makes it well-suited for open-fracture prophylaxis [15]. Its narrow spectrum also minimizes disruption of normal flora, which may preserve mucosal immune defenses and reduce selection pressure for resistant organisms [15]. In contrast, piperacillin-tazobactam is designed for broad empiric coverage in settings where polymicrobial or resistant gram-negative pathogens are suspected, such as intra-abdominal sepsis or neutropenic fever [16]. Although its spectrum includes common skin flora, its activity against gram-positive pathogens is generally less potent than that of first-generation cephalosporins [17]. Additionally, extended gram-negative and gram-negative suppression may inadvertently favor the emergence of hospital-acquired flora, including organisms that are more difficult to eradicate and that are associated with more severe systemic illness [18]. These pharmacodynamic and ecological differences may help explain why this study observed associations between cefazolin monotherapy and lower rates of osteomyelitis, sepsis, and mortality despite its narrower spectrum.

Despite broad alignment with previous work, the higher nonunion/malunion rate observed among type III fractures treated with cefazolin stands out as an important and novel finding. This pattern likely reflects residual confounding inherent to observational data. Surgeons may preferentially select piperacillin-tazobactam for more severely contaminated or complex wounds, creating unmeasured differences in soft-tissue viability, contamination level, or fracture pattern that persist after matching [9]. Additionally, TriNetX does not differentiate between type IIIA, IIIB, or IIIC injuries which are subtypes with drastically different risks of nonunion [19]. Competing risk bias may also contribute, as higher early mortality in the piperacillin-tazobactam cohort may reduce the number of patients surviving long enough to be coded with nonunion. These considerations underscore that osseous healing in severe open fractures is influenced more strongly by soft-tissue condition, vascularity, and fixation strategy than by antibiotic spectrum alone.

**Limitations**

This study employs a large, propensity matched cohort. However, several limitations warrant consideration. First, a key limitation of this analysis is the inability to account for overall injury severity. The TriNetX database does not reliably capture injury severity indices, nor does it provide granular data regarding mechanism of injury, degree of wound contamination, vascular compromise, or polytrauma burden. Consequently, injury severity could not be incorporated into the PSM model.

Because surgeons may preferentially select broader-spectrum antibiotics for patients perceived to be at higher risk, residual confounding by indication remains possible. This treatment bias could partially explain the higher rates of mortality and systemic complications such as sepsis, AKI, thromboembolic events, and mortality observed in the piperacillin-tazobactam cohort. More severely injured patients are inherently predisposed to adverse outcomes independent of antibiotic choice. Conversely, patients with seemingly less severe injuries may have been more likely to receive cefazolin, potentially contributing to improved outcomes in that cohort. Similarly, the increased nonunion/malunion rate observed in type III fractures treated with cefazolin may reflect heterogeneity in injury complexity that is not fully captured by Gustilo-Anderson classification alone.

Furthermore, another limitation relates to the characterization of antibiotic exposure within the TriNetX database. The dataset captures antibiotic administration but does not provide granular information regarding dosing, duration of therapy, or sequential antibiotic regimens. As a result, it is possible that additional antibiotics were introduced after the initial prophylactic regimen, which could introduce heterogeneity in antimicrobial exposure that cannot be fully captured within this dataset. Although all patients received antibiotics within 24 hours, precise timing of administration was not captured. Given the importance of early antibiotic delivery (ideally within 3 hours), this remains a potential confounder. Second, variations in irrigation and debridement protocols, use of antiseptic solutions, and adjunctive measures (e.g., vancomycin powder, antibiotic beads) were not standardized or consistently reported.

Although initial encounter diagnosis codes were used to preferentially capture index injury episodes, administrative coding cannot fully exclude the possibility that some patients had prior procedures, staged surgical management, or infections treated at outside institutions before presentation within the TriNetX network. As such, some degree of misclassification related to prior treatment or staged care cannot be entirely excluded. Certain postoperative complications were identified using administrative diagnosis codes, which may introduce outcome misclassification. Osteomyelitis coding may reflect suspected or treated infections, rather than microbiologically confirmed cases, and coding practices may vary across institutions. Similarly, the diagnosis of fracture nonunion or malunion often depends on radiographic interpretation and clinical judgement, which may not be consistently captured through administrative coding, potentially leading to underreporting or variability in these outcomes.

Additionally, although PSM adjusts for measured confounders, residual unobserved confounding remains possible. Surgeons may have selected broader regimens in response to intraoperative findings not coded in claims data. Furthermore, potential confounding factors like variability in post-operative care could not be fully captured. This study also utilizes the TriNetX database, which uses coding data at multiple institutions. The inherent variability in data collection methods, coding practices, and rigor across these diverse sites can introduce bias and potentially impact the generalizability of these findings. Sensitivity analyses using alternative PSM methods (e.g. varying caliper widths, matching ratios, or propensity score weighting) could further strengthen robustness. However, these analyses were not feasible with the constraints of the TriNetX platform available to the authors. This study therefore relied on SMDs to confirm post-match covariate balance and evaluated consistency of associations across Gustilo-Anderson subgroups and at both 90-day and 1-year follow-up as internal checks of robustness.

As with most large, retrospective dataset studies, this work is observational in nature and therefore cannot establish causality. However, by using matched cohorts and analyzing the largest dataset to date, this study provides evidence that challenges the prevailing assumption that broader-spectrum antibiotic coverage leads to improved outcomes.

**CONCLUSION**

This study supports the continued use of cefazolin as a first-line prophylactic antibiotic in open lower extremity fractures. While antibiotics with broader coverage may be considered in type III open fractures, this has not been fully born out in the literature. If an additional antibiotic is given, it should be administered in addition to cefazolin, not in place of it. Special considerations should be made for populations with high levels of MRSA colonization or injury-specific factors such as farm or soil exposure (necessitating anaerobic or atypical coverage) or freshwater exposure (necessitating *Pseudomonas* coverage). While residual confounding by injury severity cannot be excluded given the inherent limitations of large, federated databases, the consistency of the observed associations across multiple outcomes supports cefazolin as the primary prophylactic agent in most open lower extremity fractures. Thus, in the majority of patients, early cefazolin antibiotic prophylaxis maximizes efficacy, minimizes harm, and supports responsible antibiotic stewardship.

**REFERENCES**

1. Dheenadhayalan J, Nagashree V, Devendra A, et al (2023) Management of open fractures: A narrative review. J Clin Orthop Trauma 44:102246. https://doi.org/10.1016/j.jcot.2023.102246

2. Morgan D, Lutnick E, Mutty C, Anders M (2024) Multidisciplinary standardized protocol decreases time to antibiotic administration for open fractures. Eur J Orthop Surg Traumatol Orthop Traumatol 35:23. https://doi.org/10.1007/s00590-024-04141-z

3. Heiman E, Delaune J, Hong IS, et al (2024) Maximizing Adherence and Minimizing Time to Antibiotics: A Multidisciplinary Institutional Trauma Bay Protocol for Single Antibiotic Prophylaxis in Open Fractures. J Orthop Trauma 38:313–319. https://doi.org/10.1097/BOT.0000000000002805

4. Kim PH, Leopold SS (2012) In brief: Gustilo-Anderson classification. [corrected]. Clin Orthop 470:3270–3274. https://doi.org/10.1007/s11999-012-2376-6

5. American Academy of Orthopaedic Surgeons (2022) Prevention of Surgical Site Infections After Major Extremity Trauma: Evidence-Based Clinical Practice Guideline. American Academy of Orthopaedic Surgeons (AAOS), Rosemont, IL

6. Appelbaum RD, Farrell MS, Gelbard RB, et al (2024) Antibiotic prophylaxis in injury: an American Association for the Surgery of Trauma Critical Care Committee clinical consensus document. Trauma Surg Acute Care Open 9:e001304. https://doi.org/10.1136/tsaco-2023-001304

7. O’Connell CR, Kooda KJ, Sawyer MD, et al (2022) Evaluation of Piperacillin-Tazobactam for Antibiotic Prophylaxis in Traumatic Grade III Open Fractures. Surg Infect 23:41–46. https://doi.org/10.1089/sur.2021.122

8. Long DR, Cifu A, Salipante SJ, et al (2024) Preventing Surgical Site Infections in the Era of Escalating Antibiotic Resistance and Antibiotic Stewardship. JAMA Surg 159:949–956. https://doi.org/10.1001/jamasurg.2024.0429

9. Paterno A, Nash A, Lauck B, et al (2025) A Comparison of Piperacillin-Tazobactam With Conventional Antibiotics for Prophylaxis in Lower Extremity Type III Open Fractures. Cureus 17:e90890. https://doi.org/10.7759/cureus.90890

10. Patanwala AE, Radosevich JJ, Meshay I, et al (2019) Cefazolin Monotherapy Versus Cefazolin Plus Aminoglycosides for Antimicrobial Prophylaxis of Type III Open Fractures. Am J Ther 28:e284–e291. https://doi.org/10.1097/MJT.0000000000001121

11. Lin CA, Kobes T, Kholodovsky E, et al (2025) Outcomes Associated with Choice of Prophylactic Antibiotics in Open Fractures. J Bone Joint Surg Am 107:19–27. https://doi.org/10.2106/JBJS.24.01123

12. Nguyen T-L, Collins GS, Spence J, et al (2017) Double-adjustment in propensity score matching analysis: choosing a threshold for considering residual imbalance. BMC Med Res Methodol 17:78. https://doi.org/10.1186/s12874-017-0338-0

13. Stein E, Hüser M, Amirian ES, et al (2025) TriNetX Dataworks-USA: Overview of a Multi-Purpose, De-Identified, Federated Electronic Health Record Real-World Data and Analytics Network and Comparison to the US Census. Pharmacoepidemiol Drug Saf 34:e70198. https://doi.org/10.1002/pds.70198

14. Hayward D, Nammour MA (2025) Orthopedic and Podiatry Perioperative Antibiotics. In: StatPearls. StatPearls Publishing, Treasure Island (FL)

15. Bates TJ, Burgess MB, Garcia GR, et al (2024) Intravenous Cefazolin Achieves Sustained High Interstitial Concentrations in Open Lower Extremity Fractures. Clin Orthop 482:375–383. https://doi.org/10.1097/CORR.0000000000002808

16. Perry CM, Markham A (1999) Piperacillin/tazobactam: an updated review of its use in the treatment of bacterial infections. Drugs 57:805–843. https://doi.org/10.2165/00003495-199957050-00017

17. Beganovic M, Cusumano JA, Lopes V, et al (2019) Comparative Effectiveness of Exclusive Exposure to Nafcillin or Oxacillin, Cefazolin, Piperacillin/Tazobactam, and Fluoroquinolones Among a National Cohort of Veterans With Methicillin-Susceptible Staphylococcus aureus Bloodstream Infection. Open Forum Infect Dis 6:ofz270. https://doi.org/10.1093/ofid/ofz270

18. Tamma PD, Heil EL, Justo JA, et al (2024) Infectious Diseases Society of America 2024 Guidance on the Treatment of Antimicrobial-Resistant Gram-Negative Infections. Clin Infect Dis ciae403. https://doi.org/10.1093/cid/ciae403

19. Westgeest J, Weber D, Dulai SK, et al (2016) Factors Associated With Development of Nonunion or Delayed Healing After an Open Long Bone Fracture: A Prospective Cohort Study of 736 Subjects. J Orthop Trauma 30:149–155. https://doi.org/10.1097/BOT.0000000000000488
